# Estetrol Is Safe and Well Tolerated during Treatment of Hospitalized Men and Women with Moderate COVID-19 in a Randomized, Double-Blind Study

**DOI:** 10.3390/jcm12123928

**Published:** 2023-06-08

**Authors:** Jean Michel Foidart, Krzysztof Simon, Wulf H. Utian, Franck Mauvais-Jarvis, Jonathan Douxfils, Graham Dixon, Philip Barrington

**Affiliations:** 1 Mithra Pharmaceuticals, 4000 Liège, Belgium; gdixon@mithra.com; 2 Department of Obstetrics and Gynecology, University of Liège, 4000 Liège, Belgium; 3 Department of Infectious Diseases and Hepatology, Wrocław Medical University, 51149 Wrocław, Poland; krzysimon@gmail.com; 4 Department of Reproductive Biology, Case Western Reserve Medical School, Cleveland, OH 44106, USA; wulf@utianllc.com; 5 Department of Endocrinology and Metabolism, Tulane University School of Medicine, New Orleans, LA 70112, USA; fmauvais@tulane.edu; 6 Department of Pharmacy, Namur Thrombosis and Hemostasis Center, Faculty of Medicine, University of Namur, 5000 Namur, Belgium; jonathan.douxfils@qualiblood.eu; 7 QUALIblood s.a., 5000 Namur, Belgium; 8 tranScrip Ltd., Wokingham RG41 5TP, Berkshire, UK; phil.barrington@transcrip-group.com

**Keywords:** estetrol, E4, COVID-19, estrogen, human fetal estrogen, postmenopausal, hospitalization, coagulation, thromboembolic events

## Abstract

Epidemiological data suggest that the severe acute respiratory syndrome coronavirus 2 infection rate is higher in women than in men, but the death rate is lower, while women (>50 years) on menopausal hormone therapy (MHT) have a higher survival rate than those not on MHT. Classical oral estrogen enhances the synthesis of coagulation markers and may increase the risk of thromboembolic events that are common in coronavirus disease 2019 (COVID-19). The favorable hemostatic profile of estetrol (E4) might be suitable for use in women who are receiving estrogen treatment and contract COVID-19. A multicenter, randomized, double-blind, placebo-controlled, phase 2 study (NCT04801836) investigated the efficacy, safety, and tolerability of E4 versus placebo in hospitalized patients with moderate COVID-19. Eligible postmenopausal women and men (aged ≥ 18 years old) were randomized to E4 15 mg or placebo, once daily for 21 days, in addition to the standard of care (SoC). The primary efficacy endpoint of improvement in COVID-19 (percentage of patients recovered at day 28) between the placebo and E4 arms was not met. E4 was well tolerated, with no safety signals or thromboembolic events, suggesting that postmenopausal women can safely continue E4-based therapy in cases of moderate COVID-19 managed with SoC.

## 1. Introduction

Coronavirus disease 2019 (COVID-19) is caused by severe acute respiratory syndrome coronavirus 2 (SARS-CoV-2) and has rapidly evolved into a full-blown pandemic [1]. The SARS-CoV-2 spike protein binds to angiotensin-converting enzyme 2 (ACE2) receptors, which aids viral entry [1]. ACE2 receptors are highly expressed in a number of different types of tissues, especially the pneumocyte type II cells found in the lungs. The binding of the virus to ACE2 receptors causes the downregulation of the protective effect of ACE2 and the induction of hyper-inflammation and oxidative stress, with the consequent progression of acute lung injury (ALI) and acute respiratory distress syndrome [2]. Additionally, reduction of ACE2 leads to vasoconstriction, hypertension, coagulopathy, and the induction of inflammatory reactions that together increase the risk of ALI and COVID-19 severity [3].

**Figure 1:**
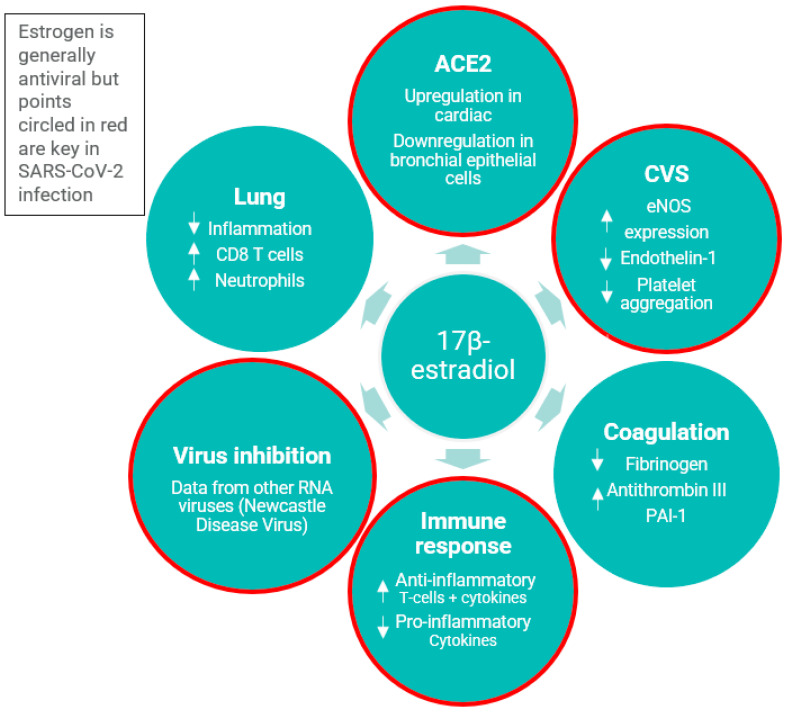
Antiviral properties of estradiol. ACE2: angiotensin-converting enzyme 2; CD8: cluster of differentiation 8; CVS: cardiovascular system; eNOS: endothelial nitric oxide synthase; PAI-1: plasminogen activator inhibitor-1; RNA: ribonucleic acid; SARS-CoV-2: severe acute respiratory syndrome coronavirus 2 [11].

Estrogens are known to have effects on the immune system and on ACE2 expression [4]. In addition to immunomodulatory effects, estrogens have been demonstrated to inhibit platelet aggregation [5]. Human fetal estrogen (estetrol, E4) is considered to have a relatively lower risk of venous thromboembolism (VTE) [6,7,8,9,10]. Thus, in postmenopausal women who require MHT while also suffering from COVID-19, a disease in which VTE and coagulation risks are increased, E4 is not believed to further increase the risk of VTE. See Figure 1 for the antiviral properties of estradiol.

Epidemiological data suggest that the SARS-CoV-2 infection rate is higher in women than in men, but the death rate is lower. Women aged 18–49 who were taking combined oral contraceptives had statistically significantly lower predicted COVID-19 symptoms and hospitalization rates. In addition, women (>50 years) on MHT had a 50% higher survival rate than those not on MHT [11,12].

In this study, male and female patients who were hospitalized with moderate COVID-19 were randomized to treatment with placebo or E4 as well as concomitant standard of care (SoC). They were then evaluated for a primary efficacy outcome measure of the percentage of patients considered recovered at day 28 (World Health Organization Ordinal Scale of Clinical Improvement [WHO OSCI] score ≤ 3; see Supplementary Table S1 for definitions).

## 2. Materials and Methods

This was an international, multicenter, randomized, double-blind, placebo-controlled, phase 2 study that investigated the efficacy, safety, and tolerability of E4 versus placebo in patients hospitalized with moderate COVID-19. 

The study was conducted at 15 centers in Belgium, Poland, and the Russian Federation. It was planned to enroll approximately 162 patients with confirmed SARS-CoV-2 infection across all countries (81 patients in the E4-treated arm and 81 patients in the placebo arm). 

Both men and postmenopausal women who met the eligibility criteria and were willing to take part in the clinical study were chosen. All patients were hospitalized due to moderate COVID-19, confirmed by reverse transcription polymerase chain reaction. A patient was defined as having moderate COVID-19 if the patient had (a) symptoms of moderate illness with COVID-19, which could include any symptom of mild illness (including fever, cough, anosmia, dysgeusia, sore throat, malaise, headache, muscle pain, gastrointestinal [GI] symptoms) or shortness of breath with exertion; (b) clinical signs suggestive of moderate illness with COVID-19, such as respiratory rate of ≥20 breaths per minute, heart rate of ≥90 beats per minute; (c) clinical frailty score of <5; (d) WHO OSCI score of 4 (hospitalized, no oxygen therapy) or 5 (hospitalized, oxygen by mask or nasal prongs). Women could not have used MHT within 1 year of study start. Patients were excluded if they were mechanically ventilated and/or were in intensive care; had any unexplained bleeding; had diagnosed protein C, protein S, or antithrombin deficiency, or any other known inherited or acquired thrombophilic abnormalities; had renal impairment, a history of endometrial hyperplasia, present or history of breast cancer, or estrogen-sensitive tumors; or were at risk of arterial or venous thrombosis/thromboembolism. 

In relation to the primary efficacy endpoint, the null hypothesis was that there was no observed difference between the E4 and placebo arms, and the alternative hypothesis was that there was an observed difference between the E4 and placebo arms. Under the assumption that 85% and 70% of patients had recovered by day 28 in the E4 and placebo arms, respectively, 162 patients were to be randomized on a 1:1 basis to provide 80% power to test the stated hypotheses at a 1-sided 0.10 alpha level. 

The study was conducted in accordance with the protocol and consensus ethical principles derived from international guidelines, including the Declaration of Helsinki, the International Council for Harmonisation Good Clinical Practice, 21 CFR 50 Protection of Human Rights, the Council for International Organizations of Medical Sciences International Ethical Guidelines, 21 CFR 56 Institutional Review Boards, and other applicable laws and regulations of the countries, Belgium, Poland, and the Russian Federation. The Independent Bioethics Committee for Scientific Research at CHU UCL Namur, site Godinne Comité d’éthique in Belgium (Ref: 249/2020); the Bioethics Committee at the Lower Silesian Medical Chamber in Poland (Ref: 26/09/2020), and the Department for State Regulation of the Circulation of Medicines of the Ministry of Health of the Russian Federation (Ref: 723) approved the study.

The primary efficacy endpoint for this study was improvement in COVID-19 between the placebo and E4 arms, as measured by the percentage of patients recovered at day 28. Recovery was defined as reaching a score of ≤3 on the WHO OSCI (0–10 scale). The safety endpoints were type, frequency, and severity of adverse events (AEs), including treatment-emergent AEs (TEAEs), serious AEs, AEs of special interest (AESIs), serious adverse reactions, and suspected unexpected serious adverse reactions; type and frequency of laboratory abnormalities; and vital signs and physical examination. The laboratory safety monitoring included the following: hematology, clinical chemistry, ferritin, C-reactive protein, and coagulation markers (prothrombin time/international normalized ratio, D-dimers, fibrinogen, von Willebrand factor, fibrin monomers, protein C, protein S, activated partial thromboplastin time, antithrombin, plasminogen activator inhibitor type 1, and tissue plasminogen activator).

Patients were randomized by an interactive voice/web response system (IXRS). The randomization was stratified by gender and based on whether patients were taking antiviral treatment for COVID-19 (e.g., remdesivir). The study center personnel, patients, contract research organization, and sponsor were blinded to the study treatment, which was supplied in blinded packaging. E4 and placebo tablets were identical in appearance and were packaged and labeled identically to ensure that the treatment was masked. Each box of medication was assigned a unique code. On randomization, the IXRS allocated a code for a box containing the appropriate treatment and the pharmacist dispensed the box to the patient. Hence, the pharmacist, site staff, and patients were blinded to the treatment allocation.

All patients were randomized to treatment with either E4 15 mg or placebo, administered once daily, orally, for 21 consecutive days, at approximately the same time each day. Patients who were discharged from the hospital during the treatment phase completed the study treatment at home. Study treatment was stopped if the patient was intubated or unable to swallow the tablets. All patients received SoC at the hospital where they were treated. Because of the increased risk of VTE, heparin was administered to all patients, provided they were not taking other anticoagulants as part of their usual medication. The anticoagulant therapy prescribed as part of this study was low-molecular-weight heparin (used for prophylaxis against deep venous thrombosis) for a minimum of 21 days. Laboratory tests for measurement of coagulation markers were performed using blood samples collected from hospitalized patients on treatment days 1, 3, 7, 10, 14, and at the end of treatment.

The primary endpoint of recovery by day 28 was analyzed via stratified logistic regression modeling. Randomized treatment was included as a class factor, and the analysis was stratified for the randomization stratification factors. The treatment effect was extracted from the model as the odds ratio for E4:placebo, along with the associated 95% 2-sided confidence interval (CI) and 1-sided *p*-value. Statistical analyses were not planned for individual baseline disease and demographic characteristics, nor for individual TEAEs. The baseline was used as a covariate in all analyses. The primary endpoint was related to the percent of patients who had recovered from COVID-19, and, therefore, a change from baseline analysis was not appropriate.

## 3. Results

**Figure 2:**
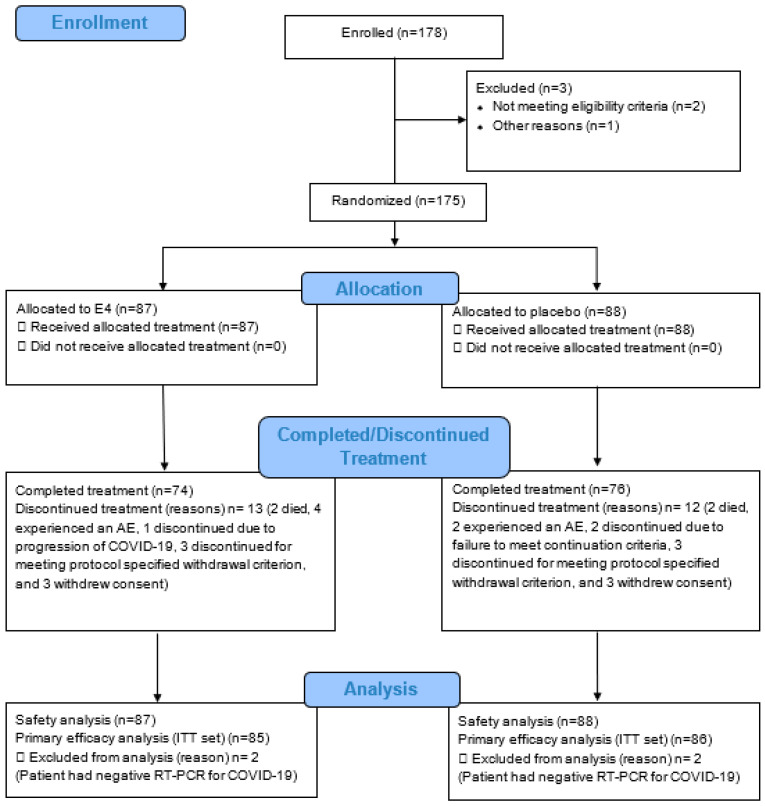
Disposition of patients. AE: adverse event; COVID-19: coronavirus disease 2019; E4: estetrol; ITT: intention-to-treat; n: number of patients; RT-PCR: reverse transcription polymerase chain reaction.

A total of 175 patients with confirmed SARS-CoV-2 infection who were hospitalized with moderate COVID-19 (per WHO OSCI scores) were enrolled between November 2020 and May 2021. The study comprised 175 patients (E4: 54 men; 33 women/placebo: 54 men; 34 women), aged between 20 and 88 years, who were randomized and treated. The minimum age of female patients enrolled was 54 years, and that of male patients enrolled was 20 years. All 175 treated patients were analyzed for safety, and 171 patients (E4: 53 males; 32 females/placebo: 53 males; 33 females) were analyzed for the primary efficacy endpoint (Figure 2). 

The demographic and baseline disease characteristics were similar for the two treatment arms, as summarized in Table 1.

All the patients in this study were Caucasian. The mean age was similar for the E4 and placebo arms (61.5 and 62.2 years, respectively) and 61.9 years overall. The mean age of male and female patients in both treatment arms was comparable (E4: 56.5 years [men]; 69.6 years [women]/placebo: 59.3 years [men]; 66.8 years [women]). Both treatment arms had mean body mass index values of approximately 29 kg/m^2^ and a similar distribution of WHO OSCI scores of 4 or 5 at baseline. In both treatment arms, ≥75% of the patients had pneumonia at the time of study enrollment, and 39% were taking antiviral medications. A high proportion of patients had comorbidities considered to be risk factors for patients with COVID-19, including a history of cardiac disorders in more than half of the patients (≥50%) in both treatment arms, and high incidences of baseline diabetes (14.9% and 19.3% in the E4 and placebo arms, respectively) and lipid abnormalities (18.4% and 12.5% in the E4 and placebo arms, respectively).

Imbalances between the two treatment arms were most prominent for respiratory disorders (E4 seven [8.0%] patients vs. placebo three [3.4%] patients) and lipid abnormalities (E4 16 [18.4%] patients vs. placebo 11 [12.5%] patients). The number of patients with a medical history of elevated cholesterol was similar in the two treatment arms (eight and seven in the E4 and placebo arms, respectively). This was also true for dyslipidemia (three and two in the E4 and placebo arms, respectively). Patients with a medical history of hyperlipidemia were higher in the E4-treated arm (four versus one in the E4 and placebo arms, respectively). One patient in the E4-treated arm suffered from hypertriglyceridemia, and one patient in the placebo arm suffered from a lipid metabolism disorder.

Asthma is a known risk factor for a variety of respiratory infections, including COVID-19. Six patients in the E4-treated arm suffered from asthma, as opposed to one patient in the placebo arm. In addition, one patient in the E4 arm suffered from a combination of asthma and chronic obstructive pulmonary disease. One patient in each treatment arm suffered from a combination of chronic bronchitis and chronic obstructive pulmonary disease. The only patient who had a medical history of chronic bronchitis in isolation was in the placebo arm.

Treatment exposure was different between the two arms, with twice as many patients in the E4 arm receiving three or fewer days of treatment compared to placebo (eight (9.2%) vs. four (4.5%)), while other lengths of exposure were similar (Table 2). 

While the numbers were small, the proportion of patients who recovered by day 7 was slightly greater in the E4 arm compared with the placebo arm (4.7% versus 2.3%, respectively) (Table 3). At day 14, the percentage of patients who recovered was similar between the E4 and placebo arms. At days 21 and 28, the percentage of patients who recovered in the placebo arm was higher than in the E4 arm. 

At day 28, 70 (82.4%) patients in the E4 arm and 79 (91.9%) patients in the placebo arm (odds ratio [OR] 0.41, 95% CI 0.16–1.07) were defined as recovered (≤3 WHO OSCI score), as summarized in Table 4. Irrespective of gender or antiviral intake, initiation of E4 after symptom onset had no treatment effect compared with placebo; therefore, the study failed to meet the primary endpoint.

The proportion of patients with all-cause mortality during the study is presented in Table 5. There were more patients with all-cause mortality at day 14, end of treatment, and end of study in the E4 arm versus the placebo arm.

Treatment with E4 was well tolerated in patients with moderate COVID-19. The frequency and types of TEAEs were similar in the E4 and placebo arms, as summarized in Table 6. In the E4 arm, four patients experienced an AE (respiratory failure [n = 3] and pulmonary embolism [n = 1]) and three patients in the placebo arm (respiratory failure, pulmonary embolism, and peripheral embolism [one patient each]) that led to discontinuation of study treatment, and five patients experienced an AESI (pulmonary embolism; three in the E4 arm, two in the placebo arm). Of all the reported serious events, only two were considered by the study investigators to have a relationship to the study treatment, and both were in the placebo arm. Gastrointestinal disorders were more common in the E4-treated arm versus the placebo arm (n = 7 [13.0%] versus n = 4 [7.4%]) in male patients. The GI side effects reported in this study in male patients who received E4 included dyspepsia (n = 2 [3.7%]), upper abdominal pain, diarrhea, nausea, duodenal ulcer, and intestinal hemorrhage (n = 1 [1.9%] for each of these AEs).

A total of eleven deaths were reported during the study, eight in the E4 treatment arm and three in the placebo arm (Table 7). Ten of the eleven deaths were associated with COVID-19 progression. None of the deaths were considered to be related to the study’s treatment. Five patients developed respiratory failure in the E4 treatment arm, with two of these patients receiving only two doses of E4, one patient receiving three doses, one patient receiving six doses, and one patient receiving twenty-one doses. None of the deaths in these five patients were considered to be related to E4 treatment.

One patient’s condition deteriorated, developing a multi-organ dysfunction syndrome after receiving only one dose of E4. Septic shock, a known complication of COVID-19 infection, occurred in two patients, one in each treatment arm. One patient in the placebo-treated arm suffered from endotoxic shock. A single case of pneumonia occurred in the placebo arm.

One patient developed peripheral arterial thrombosis after receiving twenty-one doses of E4. The thrombus occurred in the femoral popliteal artery, and the patient had a prior history of a popliteal aneurysm in this artery, which may have contributed to the formation of the thrombus. No thromboembolic events were considered to be related to treatment with E4. No treatment differences in venous or thromboembolic events were observed, and there was no obvious discrepancy in the frequency of events between the study arms (Table 8). 

## 4. Discussion

This was a multicenter, randomized, placebo-controlled study designed to evaluate the safety and efficacy of an estrogen treatment, E4, in patients who were hospitalized with moderate COVID-19. 

The design of the study was limited due to the unfamiliar pathophysiology of the disease course and the lack of universally agreed primary outcome variables in the regulatory and academic communities (for primary endpoints in other clinical studies in COVID-19 research, see Supplementary Table S2). As there were two levels of stratification (gender and concomitant antiviral treatment for COVID-19) and due to the nature of studies in COVID-19, it was not possible to randomize patients in blocks. Despite this, the patient treatment arms were relatively balanced within the centers, with the exception of length of study treatment, where more patients in the E4 arm stopped treatment within the first three days due to deterioration. Due to the nature of the pandemic, recruitment was influenced by many factors, including the workload at the site. During the peaks of COVID-19 disease, sites had to focus on treating patients instead of entering them into the study. Additionally, multiple changes in the regulatory guidance for both study design and conduct occurred during study implementation, leading to multiple amendments to the protocol. In addition, the SoC varied across countries; for example, patients in Russia could receive umifenovir as antiviral therapy, whereas this was not prescribed in Poland and Belgium. Other treatments, such as the use of dexamethasone, also varied. All patients in this study received SoC for COVID-19, which included anticoagulants. One of the most marked differences in the arms was the treatment exposure, with twice as many patients in the E4 arm receiving three or fewer days of treatment compared to placebo (eight (9.2%) vs. four (4.5%)), and this coincided with more patients in the E4 arm deteriorating rapidly over the first 48 h of the study compared to the placebo arm. Patients with a medical history of hyperlipidemia were higher in the E4 treatment arm (four versus one in the E4 and placebo arms, respectively). Six patients in the E4-treated arm had a medical history of asthma, as opposed to one patient in the placebo arm. 

The primary efficacy assessment did not show a difference in the percentage of patients who had recovered by day 28 following treatment with E4 compared with placebo. However, the comparison of the percentage of patients recovering (discharge) demonstrated that the E4 arm was similar to the placebo arm up to day 14 (see Supplementary Table S2). Of the studies of registered drugs made available for treatment of hospitalized patients with COVID-19 by the FDA, only the RECOVERY study used the percentage of patients discharged at day 28 as an outcome. E4 compared to placebo results demonstrated that percentages discharged were 82% (70/85) compared to 92% (79/86), respectively, while patients in the RECOVERY study demonstrated 78% discharged in the baricitinib plus SOC arm compared to 80% on SOC alone in over 8000 patients, and for tocilizumab plus SOC, 57% compared to 50% on SOC alone in over 4000 patients. The tocilizumab study was completed earlier in the pandemic when the disease had a longer and more severe trajectory, which may be one reason why discharge at day 28 was less reliable as an outcome [14,15,16,17].

All-cause mortality at the end of the treatment (day 28) was six patients (7.1%) on E4 and three (3.5%) on placebo. A mortality rate of 3.5% in the placebo arm is lower than any comparative study (range 9–35%) that measured all-cause mortality at day 28 (in Supplementary Table S2). Results for individual treatments show conflicting results. For example, for remdesivir, the Discovery and Solidarity studies show little difference in 28-day mortality compared to the Adaptive COVID-19 Treatment Trial (ACCT1) (Supplementary Table S2). 

Both recovery at day 28 and all-cause mortality outcomes in the study were almost certainly distorted by the imbalance in patients between the E4 and placebo arms who were withdrawn from the study during the first three days of treatment due to marked deterioration. 

There were no safety or tolerability concerns reported following treatment with E4 for 28 days in this study. There were no major differences in TEAEs reported between the E4 and placebo arms. 

A total of eleven deaths were reported during the study, eight in the E4 treatment arm and three in the placebo arm. Of the eight deaths, the difference between the two arms was in patients who died of respiratory failure and multi-organ disfunction, which accounted for six of the eight deaths on E4. Four of the deaths in the E4 arm were patients who had received ≤three doses of E4. None of the deaths were considered to be related to the study’s treatment. The most likely explanation for the patients developing respiratory failure was a consequence of COVID-19 infection. The majority of deaths were seen at one study center. An independent audit of this study center was conducted, and the findings of this audit stated that it is likely that the discrepancy was due to more of the patients with severe disease being randomly allocated to receive E4 treatment at this site. 

The rates of venous/arterial thromboembolic events were similar in the E4 and placebo arms (venous events occurred in three E4 patients and two placebo patients, and arterial events occurred in one E4 patient and two placebo patients). No thromboembolic events were considered to be related to treatment with E4. No treatment differences in venous or arterial thromboembolic events were observed, and there was no obvious discrepancy in the frequency of events between the study arms. The evaluation of inflammatory and safety biomarkers is an ongoing investigation and will be reported at a later date. 

In addition to the referenced epidemiological studies suggesting a potential protective effect of estrogens on COVID-19 mortality [11,12,18], a recent paper by Yoshida et al. [19] reported MHT to be marginally associated with a lower risk of mortality and significantly associated with a lower risk of prolonged hospital stay among inpatient women in a US national COVID-19 cohort. Various clinical studies were scheduled to investigate the impact of estrogen on COVID-19 progression and mortality (NCT04359329, NCT04865029, and NCT04539626), but only one investigated the treatment of hospitalized COVID-19. No published data are available yet. For study NCT04865029, while the number of patients recruited was small (10:5 per arm) and, therefore, not statistically powered, the duration of hospital stay appeared shorter in the active treatment arm than in the placebo arm (7.2 days [SD 5.18] versus 10.2 days [SD 7.53], respectively). Refer to Supplementary Table S3 for estrogen studies in COVID-19 and Supplementary Table S2 for other interventional studies in COVID-19.

This was the first fully recruited, randomized, placebo-controlled study to evaluate the safety and efficacy of an estrogen treatment, E4, in patients who were hospitalized with moderate COVID-19. The findings of this study show that there was no apparent effect of E4 in the treatment of moderate COVID-19. However, the limitations discussed above, especially in the context of other similarly sized studies in moderate COVID-19 patients conducted during the pandemic, suggest that showing a treatment effect was challenging. The fact that E4 in this study did not show a treatment benefit may have been related to the disease severity of the patients studied or to commencing E4 treatment relatively late during the patient’s infection. E4 might have a better likelihood of success in less severe settings, such as prevention of worsening of symptoms in mild COVID-19 or prophylactic treatment of patients at risk, the latter being more akin to the suggested protective effect of MHT in ameliorating the symptoms of COVID-19 in postmenopausal women. E4 was well tolerated, with no safety signals or deleterious effects on coagulation markers or thromboembolic events. 

## 5. Conclusions

This is the first study to report the results of estrogen treatment in hospitalized patients with COVID-19. No treatment effect of E4 compared with placebo was shown for the intention-to-treat (ITT) primary analysis of recovery at day 28, nor for admission to the ICU or all-cause mortality. E4 appears to be well tolerated, with no apparent safety signals for E4 in post-menopausal women or men suffering COVID-19. There is no indication that E4 had any deleterious effect on coagulation markers or thromboembolic events. It can be concluded that female patients who are receiving E4 MHT would not need to discontinue this treatment should they be hospitalized with moderate COVID-19 while on SoC anticoagulation therapy. The study has generated valuable biomarker information that is still being evaluated. Estrogens may play a role in the treatment of COVID-19, and several groups are conducting studies in men and women, including in the home setting where there is less impact on outcome from concomitant antivirals and anti-inflammatories.

## Figures and Tables

**Table 1:** Summary of demographics and baseline characteristics for patients with moderate COVID-19 infection (randomized population).

Variable	E4 (N = 87)	Placebo (N = 88)	Total (N = 175)
Sex, n (%)			
Male	54 (62.1)	54 (61.4)	108 (61.7)
Female	33 (37.9)	34 (38.6)	67 (38.3)
Race, n (%)			
White	87 (100)	88 (100)	175 (100)
Age (years) (overall)			
Mean (SD)	61.5 (12.7)	62.2 (11.6)	61.9 (12.1)
Median	62.0	63.0	62.0
Q1, Q3	53.0, 70.0	58.5, 69.5	56.0, 70.0
Min, Max	30, 88	20, 85	20, 88
Age (years) (men)			
Mean (SD)	56.5 (12.32)	59.3 (12.86)	57.9 (12.61)
Median	56.5	62.0	59.0
Q1, Q3	47.0, 64.0	51.0, 69.0	48.5, 67.5
Min, Max	30, 82	20, 84	20, 84
Age (years) (women)			
Mean (SD)	69.6 (8.34)	66.8 (7.39)	68.2 (7.95)
Median	68.0	65.5	67.0
Q1, Q3	63.0, 73.0	61.0, 72.0	62.0, 73.0
Min, Max	54, 88	54, 85	54, 88
BMI (kg/m^2^)			
Mean (SD)	29.1 (5.3)	28.8 (5.1)	29.0 (5.2)
Median	27.4	27.8	27.8
Q1, Q3	25.1, 33.2	25.3, 32.0	25.2, 32.4
Min, Max	19.8, 43.3	19.7, 48.9	19.7, 48.9
Clinical frailty score			
Mean (SD)	2.9 (1.0)	2.8 (0.9)	2.9 (1.0)
Median	3.0	3.0	3.0
Q1, Q3	2.0, 4.0	2.0, 3.0	2.0, 3.0
Min, Max	1, 5	1, 5	1, 5
Pneumonia, n (%)			
Yes	66 (75.9)	69 (78.4)	135 (77.1)
No	21 (24.1)	18 (20.5)	39 (22.3)
Missing data ^a^	-	1 (1.1)	1 (0.6)
Antiviral medication, n (%) ^b^			
Yes	35 (40.2)	33 (37.5)	68 (38.9)
No	50 (57.5)	53 (60.2)	103 (58.9)
Missing data ^c^	2 (2.3)	2 (2.3)	4 (2.3)
WHO OSCI score, n (%) ^d^			
4	15 (17.2)	14 (15.9)	29 (16.6)
5	71 (81.6)	74 (84.1)	145 (82.9)
6 ^e^	1 (1.1)	0	1 (0.57)
Medical history—key known COVID risk factors, n (%)			
Cardiac disorders	44 (50.6)	51 (58.0)	95 (54.3)
Diabetes	13 (14.9)	17 (19.3)	30 (17.1)
Lipid abnormalities	16 (18.4)	11 (12.5)	27 (15.4)
Respiratory disorders	7 (8.0)	3 (3.4)	10 (5.7)

BMI: body mass index; COVID-19: coronavirus disease 2019; E4: estetrol; Min: minimum; Max: maximum; N: total number of patients; n: number of patients; OSCI: Ordinal Scale of Clinical Improvement; Q: quarter; RT-PCR: reverse transcription polymerase chain reaction; SD: standard deviation; WHO: World Health Organization. ^a^ The pneumonia status for Patient POL-012-0005 was provided after baseline and therefore not included in the data listing. ^b^ The antiviral medications used were remdesivir (most common), favipiravir, amantadine hydrochloride, imidazolyl ethanamide pentandioic acid, riamilovir, and umifenovir. ^c^ The use of antiviral medication was not captured for two patients in the E4 arm and two patients in the placebo arm at baseline; these patients were withdrawn due to a negative RT-PCR after randomization. ^d^ WHO OSCI: World Health Organization Ordinal Scale for Clinical Improvement. A severity grading for COVID-19. WHO Working Group. Lancet Infect Dis. 2020 [13]. ^e^ One patient recorded a WHO OSCI score of 5 at screening that had deteriorated to 6 at the time of baseline.

**Table 2:** Summary of treatment exposure (safety population).

Number of Patients Receiving a Range of Doses, n (%)			
	E4 (N = 87)	Placebo (N = 88)	Total (N = 175)
≤3	8 (9.2)	4 (4.5)	12 (6.9)
>3 to ≤7	2 (2.3)	2 (2.3)	4 (2.3)
>7 to ≤14	3 (3.4)	5 (5.7)	8 (4.6)
>14 to 21	74 (85.1)	77 (87.5)	151 (86.3)

E4: estetrol; min: minimum; max: maximum; N: total number of patients; n: number of patients; SD: standard deviation.

**Table 3:** Recovered patients (WHO OSCI Score ≤ 3) at days 7, 14, 21, and 28 (ITT population).

Category	E4 15 mg (N = 85) n (%)	Placebo (N = 86) n (%)
Day 7	4 (4.7%)	2 (2.3%)
Day 14	51 (60.0%)	52 (60.5%)
Day 21	62 (72.9%)	76 (88.4%)
Day 28	70 (82.4%)	79 (91.9%)

E4: estetrol; N: number of patients in the treatment arm; n: number of patients; WHO OSCI: World Health Organization Ordinal Scale of Clinical Improvement.

**Table 4:** Patients with a WHO OSCI score ≤3 at day 28 (primary efficacy endpoint) with background antiviral intake (ITT population).

Category	E4 15 mg (N = 85) n (%)	Placebo (N = 86) n (%)	Odds Ratio ^a^	
			Estimate	95 % 2-Sided CI
All	70 (82.4%)	79 (91.9%)	0.41	0.16–1.07
Males				
Antiviral medication at baseline	19/24 (79.2%)	19/21 (90.5%)		
No antiviral medication at baseline	25/29 (86.2%)	28/32 (87.5%)		
Females				
Antiviral medication at baseline	10/11 (90.9%)	12/12 (100.0%)		
No antiviral medication at baseline	16/21 (76.2%)	20/21 (95.2%)		

CI: confidence interval; COVID-19: coronavirus disease 2019; E4: estetrol; ITT: intention-to-treat; N: number of patients in the treatment arm; n: number of patients; WHO OSCI: World Health Organization Ordinal Scale of Clinical Improvement. Note: Under the hypothesis that E4 would be superior to placebo and the one-sided logistic regression model applied, a *p*-value was not calculable. ^a^ Odds ratio from the logistic regression model with randomized treatment included as a class factor, baseline WHO (0–10) score as a class factor, and age included as covariates and stratified for the randomization stratification factors of gender and antiviral treatment for COVID-19 at baseline at a given timepoint.

**Table 5:** Cumulative proportion of patients with all-cause mortality during the study (ITT population).

Category	E4 15 mg (N = 85) n (%)	Placebo (N = 86) n (%)	Total (N = 171)
Day 14	3 (3.5%)	2 (2.3%)	5 (2.9%)
End of Treatment	5 (5.9%)	3 (3.5%)	8 (4.7%)
End of Study (Day 28)	6 (7.1%) ^a^	3 (3.5%)	9 (5.3%)

E4: estetrol; ITT: intention-to-treat; N: number of patients in the study arm; n: number of patients. ^a^ There were two additional deaths in the E4 arm post-end-of-study (one on day 32 due to progressive disease and one on day 42 [unrelated]).

**Table 6:** Summary of treatment-emergent adverse events (safety population).

Adverse Event Category	E4 15 mg (N = 87) n (%) E	Placebo (N = 88) n (%) E
Overall		
Any TEAE	55 (63.2) 122	47 (53.4) 123
TEAEs leading to discontinuation of study treatment	4 (4.6) 4	3 (3.4) 3
Adverse events of special interest (AESI) ^a^	3 (3.4) 3	2 (2.3) 2
Serious adverse events	11 (12.6) 12	7 (8.0) 10
Serious adverse event with outcome of death	8 (9.2) 8	3 (3.4) 3
Men		
Any TEAE	34 (63.0) 85	25 (46.3) 71
TEAEs leading to discontinuation of study treatment	3 (5.6) 3	3 (5.6) 3
Adverse events of special interest (AESI) ^a^	2 (3.7) 2	2 (3.7) 2
Serious adverse events	5 (9.3) 5	5 (9.3) 7
Serious adverse event with outcome of death	4 (7.4) 4	2 (3.7) 2
Women		
Any TEAE	21 (63.6) 37	22 (64.7) 52
TEAEs leading to discontinuation of study treatment	1 (3.0) 1	0 (0.0) 0
Adverse events of special interest (AESI) ^a^	1 (3.0) 1	0 (0.0) 0
Serious adverse events	6 (18.2) 7	2 (5.9) 3
Serious adverse event with outcome of death	4 (12.1) 4	1 (2.9) 1

AESI: adverse event of special interest; E: number of events; E4: estetrol; N: number of patients in the treatment arm; n: number of patients; TEAE: treatment emergent adverse event. ^a^ AESI were defined as any venous thromboembolism.

**Table 7:** Number of deaths with reason of death and number of doses of study treatment (safety population).

	E4 15 mg (N = 87)	Placebo (N = 88)	No. of Doses of Study Treatment
Any treatment-emergent adverse events (TEAEs) with fatal outcome	8 (9.2%)	3 (3.4%)	
Reason of death			
Respiratory failure	5 (5.7%)	0	1 patient each: 3, 6, 21 doses 2 patients had 2 doses
Multiple organ dysfunction syndrome	1 (1.1%)	0	1 dose
Septic shock	1 (1.1%)	1 (1.1%)	E4: 11 doses Placebo: 9 doses
Endotoxic shock	0	1 (1.1%)	10 doses
Pneumonia	0	1 (1.1%)	14 doses
Peripheral artery thrombosis	1 (1.1%)	0	21 doses

**Table 8:** Thromboembolic events (safety population).

Study Treatment	Age of Patient/ Gender	D-Dimer Approximate Peak Concentration (ng/mL)	Number of Doses of Study Treatment	Relationship to Study Treatment ^a^
Clinical deep vein thrombosis				
None reported				
Venous thromboembolism (pulmonary embolus)				
E4	71/female	1700	21	Not related
E4	46/male	44,200	21	Not related
E4	58/male	2800	14	Unlikely related
Placebo	76/male	6800	7	Possibly related
Placebo	43/male	15,900	6	Unlikely related
Arterial thrombosis				
E4	62/male	23,600	21	Not related
Placebo	60/female	1700	20	Not related
Placebo	67/male	7200	19	Not related

E4: estetrol. ^a^ Relationship to study treatment was determined by the investigator.

## Data Availability

The data presented in this study are available on request from the corresponding author.

## Supplementary Materials

The following supporting information can be downloaded at: https://www.mdpi.com/article/10.3390/jcm12123928/s1, Table S1: WHO Ordinal Scale for Clinical Improvement; Table S2: Current Study and Other Randomized-Controlled Design Interventions Including Hospitalized Moderate COVID 19.; Table S3: Estrogen Studies in COVID-19 Recruiting Both Female and Male Patients. Refs. [20,21,22,23,24,25,26,27,28,29,30,31,32,33,34,35] are cited in the Supplementary Materials.
