# Supplementary material for: Estetrol Is Safe and Well Tolerated during Treatment of Hospitalized Men and Women with Moderate COVID-19 in a Randomized, Double-Blind Study
Source: J Clin Med. 2023 Jun 8;12(12):3928. doi: 10.3390/jcm12123928 (PMC10299386; doi:10.3390/jcm12123928)
Supplement: Supplementary file 1 [file jcm-12-03928-s001.zip › jcm-2394135-supplementary.pdf]

Supplementary Materials

Table S1 - WHO Ordinal Scale for Clinical Improvement 1
Table S2 - Current Study and Other Randomized-Controlled Design Interventions Including Hospitalized Moderate COVID 19 2
Table S3 - Estrogen Studies in COVID-19 Recruiting Both Female and Male Patients............. 8

Table S1. WHO Ordinal Scale for Clinical Improvement

Patient State	Descriptor	Score
Uninfected	Uninfected; no viral RNA detected	0
Ambulatory, mild disease	Asymptomatic, viral RNA detected	1
	Symptomatic, independent	2
	Symptomatic, assistance needed	3
Hospitalized		
Moderate disease	Hospitalized, no oxygen therapy	4
Moderate disease	Oxygen by mask or nasal prongs	5
Severe disease	Non-invasive ventilation or high-flow oxygen	6
Severe disease	Intubation and mechanical ventilation, $pO_2/FiO_2 \geq 150$ or $SpO_2/FiO_2 \geq 200$	7
Severe disease	Mechanical ventilation $pO_2/FiO_2 < 150$ ($SpO_2/FiO_2 < 200$) or vasopressin	8
Severe disease	Mechanical ventilation $pO_2/FiO_2 < 150$ and vasopressin, dialysis, or ECMO	9
Dead	Death	10

COVID-19: Coronavirus disease 2019; ECMO: extracorporeal membrane oxygenation; FiO_2: fraction of inspired oxygen; pO2: oxygen partial pressure; RNA: ribonucleic acid; SpO_2: oxygen saturation; WHO: World Health Organization.

WHO Working Group on the Clinical Characterisation and Management of COVID-19 infection. A minimal common out-come measure set for COVID-19 clinical research (published correction appears in Lancet Infect Dis. 2020 Oct;20(10):e250). Lancet Infect Dis. 2020;20(8):e192-e197. doi:10.1016/S1473-3099(20)30483-7 [13]

Table S2. *Current Study and Other Randomized-Controlled Design Interventions Including Hospitalized Moderate COVID 19*

Study	Treatment	n	Outcomes	Results	Study Limitations
**E4**					
(current study)	E4 15 mg *versus* placebo for 21 days	87 (E4)<br>88 (placebo)	**Primary:** Percentage patients recovered at Day 28 Discharged (WHO OSCI <3)	E4: 70/85 (82.4%)<br>Placebo: 79/86 (91.9%)	
			Patients recovered by Day 14 (WHO OSCI <3)	E4: 51/85 (60.0%)<br>Placebo: 52/86 (60.5%)	
			Percentage of patients with WHO OSCI >6 at Day 28	E4: 10/85 (11.8%)<br>Placebo: 6/86 (7.0%)	
			Time to recovery defined as time to a score of ≤3 on the WHO OSCI scale	E4: 13 days (CI 95% 12-14) Placebo: 12 days (CI 95% 11-14)	
**Remdesivir**					
ACCT1 (Oct 2020) [14]	Remdesivir 200 mg IV on Day 1, then 100 mg IV once daily for up to 9 more days *versus* placebo	541 (remdesivir)<br>521 (placebo)	**Primary:** Time to clinical recovery	Remdesivir: 10 days<br>Placebo: 15 days<br>Rate ratio for recovery 1.29; 95% CI 1.12–1.49; *p*<0.001	Wide range of disease severity among patients; study not powered to detect differences within subgroups; study not powered to detect differences in mortality between arms; no data on long-term morbidity
			Improvement in clinical status at Day 15	More likely in remdesivir arm than placebo (OR 1.5; 95% CI 1.2–1.9; *p*<0.001)	
			Mortality by Day 29	Remdesivir: 11.4%<br>Placebo: 15.2%<br>HR 0.73; 95% CI, 0.52 to 1.03	
CATCO (Jan 2022) [20]	Remdesivir 200 mg IV on Day 0, then 100 mg	634 (remdesivir)<br>647 (SOC)	In-hospital mortality	Remdesivir: 18.7%<br>SOC: 22.6%<br>RR 0.83; 95% CI 0.67–1.03	Open-label study; information on comorbidities was not available for 26% of patients

	IV once daily on Days 1–9 *versus* local SOC		New need for mechanical ventilation	Remdesivir: 8%<br>SOC: 15%<br>RR 0.53; 95% CI 0.38–0.75	
			Hospital length of stay	No significant difference	
Discovery (Sep 21) [21]	Remdesivir 200 mg IV on Day 1, 100 mg IV once daily for up to 9 days *versus* SOC	429 (remdesivir)<br>428 (SOC)	**Primary:** Clinical status at Day 15	No difference between arms (OR 0.98; 95% CI 0.77–1.25; *p*=0.85)	Open-label study; 440 participants in this study also enrolled in the WHO Solidarity study
			Mortality by Day 29	Remdesivir: 8%<br>SOC: 9%	
WHO Solidarity (Oct 2022) [22]	Remdesivir 200 mg IV on Day 1, 100 mg IV once daily for up to 9 days *versus* SOC	4146 (remdesivir)<br>4129 (local SOC)	**Primary:** In-hospital mortality	Remdesivir: 14.5%<br>SOC: 15.6%<br>Rate ratio 0.91; 95% CI 0.82–1.02; *p*=0.12	Open-label study; no data on time from symptom onset to enrollment
			Initiation mechanical ventilation	Remdesivir: 14.1%<br>SOC: 15.7%<br>Rate ratio 0.88; 95% CI 0.77–1.00; *p*=0.04	
Spinner et al (Sep 2020) [23]	Remdesivir 200 mg IV on Day 1, then 100 mg IV once daily for 9 days or remdesivir 200 mg IV on Day 1, then 100 mg IV once daily for 4 days *versus* local SOC	193 (remdesivir 10 days)<br>191 (remdesivir 5 days)<br>200 (SOC)	**Primary:** Clinical status at Day 11 measured by WHO OSCI	Significantly better in 5-day remdesivir arm than in SOC arm (OR 1.65; 95% CI 1.09–2.48; *p*=0.02); no difference between 10-day remdesivir arm and SOC arm (*p*=0.18)	Open-label study
Goldman et al (May 2020) [24]	Remdesivir 200 mg IV on Day 1, then 100 mg IV once daily for 4 days *versus* remdesivir 200 mg IV on Day 1, then 100 mg IV once daily for 9 days	200 (remdesivir 10 days)<br>197 (remdesivir 5 days)	**Primary:** Clinical status at Day 14, as measured by an WHO OSCI	Proportion with clinical improvement at Day 14: 64% in 5-day remdesivir arm vs 54% in 10-day remdesivir arm (*p*=0.14)	Open-label study; lack of placebo arm; baseline imbalances in clinical status of patients in 5-day remdesivir and 10-day remdesivir arms

**Dexamethasone**					
RECOVERY (Feb 2021) [25]	Dexamethasone 6 mg IV or PO once daily plus SOC for up to 10 days or until discharge *versus* SOC alone	2104 (dexamethasone + SOC)<br>4321 (SOC)	**Primary:** All-cause mortality at 28 days	Dexamethasone: 23%<br>SOC: 26%<br>Age-adjusted rate ratio 0.83; 95% CI 0.75–0.93; $p<0.001$	Open-label study; published data did not include results for key secondary endpoints (e.g., cause-specific mortality, need for renal replacement), AEs, and key subgroups (e.g., patients with comorbidities); patients who required supplemental oxygen (but not MV) had variable severity of illness, and it is unclear whether all patients in this subgroup benefited from dexamethasone or whether benefit is restricted to those requiring higher levels of supplemental oxygen; patients aged >80 years were preferentially assigned to receive supplemental oxygen therapy (and not MV); high mortality in this study may limit the generalizability of results to populations with a lower baseline mortality
			Patients who required MV or ECMO at randomization	Dexamethasone: 29%<br>SOC: 41%<br>Rate ratio 0.64; 95% CI 0.51–0.81	
			Patients who required supplemental oxygen but not MV at randomization	Dexamethasone: 23%<br>SOC: 26%<br>Rate ratio 0.82; 95% CI 0.72–0.94	
			Patients who did not require supplemental oxygen at randomization:	Dexamethasone: 18%<br>SOC: 14%<br>Rate ratio 1.19, 95% CI 0.92–1.55	
**Baricitinib**					
RECOVERY (July 2022) [26]	Baricitinib 4 mg PO daily for 10 days or until discharge (whichever comes first) *versus* SOC	4148 (baricitinib)<br>4008 (SOC)	28-day mortality	Baricitinib: 12%<br>SOC: 14%<br>Age-adjusted rate ratio 0.87; 95% CI 0.77–0.98; $p=0.028$	Open-label study
			Discharge within 28 days	Baricitinib: 80%<br>SOC: 78%	

				Age-adjusted rate ratio 1.10; 95% CI 1.04–1.15; *p*=0.0002	
			Median time to discharge	8 days in both arms	
			Composite of MV, ECMO, or death	Baricitinib: 16%<br>SOC: 17%<br>Age-adjusted risk ratio 0.89; 95% CI 0.81–0.98; *p*=0.016	
COV Barrier (Sep 2021) [27]	Baricitinib 4 mg PO once daily for up to 14 days *versus* placebo	764 (baricitinib)<br>761 (placebo)	Clinical progression or death by Day 28	Baricitinib: 28%<br>Placebo: 31%<br>OR 0.85; 95% CI 0.67–1.08; *p*=0.18	Results from the ACTT-2 study prompted a protocol amendment limiting enrollment to participants who required baseline oxygen
			Mortality by Day 28	Baricitinib: 8%<br>Placebo: 13%<br>HR 0.57; 95% CI 0.41–0.78; *p*=0.0018	
			Mortality by Day 28 for those receiving corticosteroids at baseline	Baricitinib: 9%<br>Placebo: 14%<br>HR 0.63; 95% CI 0.45–0.89; *p*=0.017	
Kalil et al (Mar 2021) [28]	Baricitinib 4 mg PO once daily for 14 days or until discharge, plus RDV for 10 days or until discharge (n = 515), *versus* placebo plus RDV (n = 518)	515 (baricitinib plus SOC)<br>518 (placebo plus RDV)	Time to recovery by Day 28	Baricitinib: 7 days<br>Placebo: 8 days<br>Rate ratio 1.16; 95% CI 1.01–1.32; *p*=0.03	Not powered to detect difference in mortality between arms; steroids not part of SOC
			Improvement in clinical status at Day 15	Greater in baricitinib arm than placebo arm (OR 1.3; 95% CI 1.0–1.6)	
			Mortality at Day 28	Baricitinib: 5%<br>Placebo: 8%<br>HR 0.65; 95% CI 0.39–1.09	
**Tocilizumab**					
	1 weight-based dose of tocilizumab		**Primary:** 28-day all-cause mortality	Tocilizumab: 31%<br>Usual care: 35%	Arbitrary CRP ≥75 mg/L cutoff for enrollment; difficult to define

RECOVERY (May 21) [15]	(maximum 800 mg) with possible second dose *versus* usual care	2022 (tocilizumab plus SOC)<br>2094 (usual care)		Rate ratio 0.85; 95% CI 0.76–0.94; *p*=0.0028	exact subset of patients in RECOVERY cohort who were subsequently selected for secondary randomization/tocilizumab study
			Proportion discharged from hospital within 28 days	Tocilizumab: 57%<br>Usual care: 50%<br>Rate ratio 1.22; 95% CI 1.12–1.33; *p*<0.0001	
			Median time to hospital discharge	Tocilizumab: 9 days<br>Usual care: >28 days	
			Proportion not on MV at baseline who died or required MV within 28 days	Tocilizumab: 35%<br>Usual care: 42%<br>Rate ratio 0.84; 95% CI 0.77–0.92; *p*<0.0001	
COVACTA (Feb 2021) [16]	1 dose of tocilizumab 8 mg/kg with possible second dose plus SOC *versus* placebo plus SOC	294 (tocilizumab)<br>144 (placebo plus SOC)	**Primary:** Clinical status at Day 28, as measured by an OS	No significant difference between arms in clinical status at Day 28 (*p*=0.31)	Modest power to detect differences in Day 28 clinical status; more patients received corticosteroids in placebo arm than tocilizumab arm
			Median time to hospital discharge:	Tocilizumab: 20 days<br>Placebo: 28 days<br>HR 1.35; 95% CI 1.02–1.79	
			Mortality by Day 28	Tocilizumab: 20%<br>Placebo: 19%<br>*p*=0.94	
EMPACTA (Dec 2020) [29]	1 dose of tocilizumab 8 mg/kg with possible second dose, plus SOC *versus* placebo plus SOC	249 (tocilizumab plus SOC)<br>128 (placebo plus SOC)	**Primary:** Proportion who progressed to MV	Tocilizumab: 12%<br>Placebo: 19%<br>HR 0.56; 95% CI 0.33–0.97; *p*=0.04	Moderate sample size
			Median time to hospital discharge or readiness for discharge	Tocilizumab: 6.0 days<br>Placebo: 7.5 days<br>HR 1.16; 95% CI 0.91–1.48	
			All-cause mortality by Day 28	Tocilizumab: 10.4%<br>Placebo: 8.6%	

BACC Bay (Oct 2020) [30]	Tocilizumab 8 mg/kg plus usual care *versus* placebo plus usual care	161 (tocilizumab)<br>81 (placebo plus usual care)	**Primary:** Progression to MV or death by Day 28	Tocilizumab: 11%<br>Placebo: 13%<br>HR 0.83; 95% CI 0.38–1.81; p=0.64	Wide confidence intervals due to small sample size and low event rates; wide confidence intervals due to small sample size and low event rates; few patients received remdesivir or corticosteroids
			Proportion with clinical worsening of disease by Day 28	Tocilizumab: 19%<br>Placebo: 17%<br>HR 1.11; 95% CI 0.59–2.10; p=0.73	
			Median time to discontinuation of oxygen	Tocilizumab: 5.0 days<br>Placebo: 4.9 days<br>p=0.69	
REMDACTA (Nov 2021 [31]	Up to 10 days of remdesivir plus tocilizumab 8 mg/kg IV with second dose within 8–24 hours if indicated *versus* placebo	434 (tocilizumab)<br>215 (placebo)	**Primary:** Time to hospital discharge or readiness for discharge by Day 28	14 days in each arm (HR 0.97; 95% CI 0.78–1.19; p=0.74)	During the study, primary outcome changed from clinical status at Day 28 to time to hospital discharge or readiness for discharge by Day 28; imbalances in patient characteristics at baseline between arms; possible underrepresentation of patients with rapidly progressive disease
			Proportion who required MV or died by Day 28	29% in each arm; time to death not evaluable (HR 0.98; 95% CI 0.72–1.34; p=0.90)	
			Mean ordinal score for clinical status at Day 14	Tocilizumab: 2.8<br>Placebo: 2.9<br>p=0.72	
			Proportion who died by Day 28	Tocilizumab: 18%<br>Placebo: 20%	

Note: The secondary endpoints included are chosen based on their relevance to other studies.
CI: confidence interval; CRP: C-reactive protein; ECMO: extracorporeal membrane oxygenation; HR: hazard ratio; IV: intravenous; MV: mechanical ventilation; n: number of patients in the treatment arm; OR: odds ratio; RR: relative risk; SOC: standard of care; WHO OSCI: World Health Organization Ordinal Scale of Clinical Improvement.

Table S3. *Estrogen Studies in COVID-19 Recruiting Both Female and Male Patients.*

Title	EudraCT No.	Phase	Treatment	Recruitment status	Results
Estrogen patch for COVID-19 symptoms [32]	NCT04359329	2	E2 patch 100 mg/day for 7 days	2 subjects recruited	No published results
Estrogen therapy in non-severe COVID-19 patients [33]	NCT04539626	Not applica ble	Weekly EVRA patches (norelgestromin 6 mg/EE 0.60 mg) 1 patch placed every week for 21 days	44 subjects recruited	No published results
Oestrogen treatment for COVID-19 symptoms [34]	NCT04853069	2	17ß-estradiol gel 3 mg/day for 10 days	Recruitment status unknown	No published results
Estradiol and progesterone in hospitalized COVID-19 patients [35]	NCT04865029	2	E2 cypionate 5 mg/mL injection at admission and progesterone oral 200 mg/day for 5 days starting at admission	10 subjects recruited (5 subjects in each treatment arm)	One fatality was reported in the placebo treatment arm. All participants except the patient who died, achieved a WHO OSCI score of 1–2 at discharge. The duration of hospital stay was shorter in the active treatment arm than in the placebo group (7.2 days [SD 5.18] versus 10.2 days [SD 7.53], respectively)

COVID-19: Coronavirus disease 2019; E2: estradiol; EE: ethinyl estradiol; SD: standard deviation; WHO OSCI: World Health Organization Ordinal Scale of Clinical Improvement.
